# Oral L-Arginine treatment attenuates *Cryptococcus neoformans* extrapulmonary dissemination and disease progression

**DOI:** 10.1080/21505594.2025.2591455

**Published:** 2025-11-19

**Authors:** Adithap Hansakon, Pakkapong Phucharoenrak, Dunyaporn Trachootham, Suticha Kaewrattana, Siranart Jeerawattanawart, Warisraporn Tangchang, Methee Chayakulkeeree, Nasikarn Angkasekwinai, Pornpimon Angkasekwinai

**Affiliations:** a Chulabhorn International College of Medicine, Thammasat University, Pathum Thani, Thailand; b Department of Medical Technology, Faculty of Allied Health Sciences, Thammasat University, Pathum Thani, Thailand; c Institute of Nutrition, Mahidol University, Nakhon Pathom, Thailand; d Faculty of Medical Technology, Rangsit University, Pathum Thani, Thailand; e Center for Animal Research, Naresuan University, Phitsanulok, Thailand; f Division of Infectious Diseases and Tropical Medicine, Department of Medicine, Faculty of Medicine, Siriraj Hospital, Mahidol University, Bangkok, Thailand; g Research Unit in Molecular Pathogenesis and Immunology of Infectious Diseases, Thammasat University, Pathum Thani, Thailand

**Keywords:** Arginine, Metabolism, *Cryptococcus neoformans*, fungal dissemination, brain infection

## Abstract

*Cryptococcus neoformans* is an opportunistic fungal pathogen causing severe infections in immunocompromised individuals. Arginine metabolism is critical for immune regulation, but its precise role in cryptococcal pathogenesis is not well understood. In this study, we investigated systemic and tissue-specific alterations in L-arginine metabolism during pulmonary *C. neoformans* infection and evaluated L-arginine supplementation as a potential therapy using a murine model. Key assessments included fungal burden quantification, inflammatory cell and cytokine characterization, brain gene expression analysis, histological examinations, and survival studies. We found significant depletion of serum L-arginine and its downstream metabolites, accompanied by increased arginase activity in infected tissues, indicating a disrupted metabolic balance. Gene expression analysis showed distinct metabolic shifts, including upregulation of arginase-1 (Arg1) and proline metabolism genes, with concurrent suppression of nitric oxide synthase 2 (Nos2) in the brain during the late infection phase. Oral L-arginine supplementation significantly reduced fungal burdens in the brain and spleen, suggesting its effectiveness in controlling cryptococcal dissemination from the lungs. Consequently, L-arginine administration improved survival and clinical scores while also reducing brain cryptococcoma in infected mice. Mechanistically, L-arginine enhanced protective immune responses within the mouse brain, facilitated microglial-mediated clearance of *Cryptococcus*, and reduced cryptococcal invasion across brain endothelial cells *in vitro*. In summary, oral administration of L-arginine mitigates *C. neoformans* dissemination by augmenting brain’s immune response. This study provides crucial insights into arginine metabolism in cryptococcal disease progression, supporting L-arginine as a promising immunomodulatory therapy.

## Introduction

Cryptococcosis, resulting from infection by *C. neoformans*, remains a significant public health concern globally, particularly among immunocompromised individuals [1,2]. Most people are infected by this fungus through the inhalation of desiccated yeast or basidiospore from the environment associated with pigeon excreta [3]. Pulmonary infection initially occurs upon infection, presenting clinically with mild symptom or progressing to pulmonary cryptococcosis with pneumonia [3]. The infection caused by this fungus is not limited to the lung, as it has the potential to disseminate into other organs, particularly the brain. In patients with advanced HIV/AIDS, cryptococcal meningitis is recognized as a serious type of infection, with more than 152,000 mortality cases reported annually [4]. The World Health Organization (WHO) has recently underscored the importance of *C. neoformans*, which has been categorized as one of the most critical fungal pathogens due to disease’s virulence, diagnosis, and limited treatment strategies [2]. Therefore, it is critically necessary to investigate innovative protective and therapeutic strategies against cryptococcal infection.

L-arginine is an important amino acid that serves as a precursor for nitric oxide (NO) and multiple immunomodulatory metabolites such as polyamines [5–9]. L-arginine availability is regulated by two independent enzymatic pathways: arginase and inducible nitric oxide synthase (iNOS). The enzyme arginase catalyzes the conversion of L-arginine into urea, ornithine, and polyamine metabolites; however, nitric oxide synthase catalyzes the conversion of L-arginine to nitric oxide, citrulline, and other reactive nitrogen intermediates [10–12]. Consequently, L-arginine is crucial in modulating the immune response by influencing both innate and adaptive immunity [9]. Related studies indicated that elevated arginase-1 levels resulted in L-arginine depletion in various immune-related clinical conditions, such as inflammation-triggered immune dysfunction, cancer cell immune evasion, fibrosis, and immune responses to pathogen infections [13–20]. Many studies have investigated the therapeutic potential of L-arginine administration to modulate the L-arginine-arginase pathway in allergic asthma, cancer, and infectious diseases, including *Mycobacterium tuberculosis, Leishmania, Helicobacter, and Trypanosoma cruzi* infection [13–20]. Some of these studies are currently in the clinical trial phase [21–23]. Related research has shown that the expression ratio of nitric oxide synthases and arginases in macrophage changes as disease progresses during *Cryptococcus* infection [24,25]. *C. neoformans* with high virulence can stimulate high expression of arginase enzymes ( > 1000 fold) in both lung and alveolar macrophages [25,26]. However, the alteration of L-arginine metabolism and its contribution to cryptococcal infection remain uncertain.

This study sought to investigate the alteration and modification of L-arginine metabolites during *C. neoformans* infection and its influence on regulation of disease pathogenesis. Our findings revealed a marked reduction in serum L-arginine levels and its downstream metabolites throughout the late phase of *C. neoformans* infection. In *C. neoformans*-infected mice, L-arginine administration significantly reduced fungal dissemination while boosting inflammatory cytokine responses in the brain. L-arginine treatment also prolonged mouse survival, improved clinical signs of illness, and reduced brain cryptococcomas. In vitro, L-arginine supplementation of BV2 microglial cells improved cryptococcal clearance, promoted a protective M1 phenotype, and diminished cryptococcal invasion in a blood-brain barrier model. Collectively, these data highlight L-arginine’s potential as a therapeutic target for *C. neoformans* infection.

## Materials and methods

### Mice

Female BALB/c mice, aged 6–8 weeks, were acquired from Nomura Siam International Co., Ltd, Thailand, and housed in a specific pathogen-free environment at the animal facility of Thammasat University. At the designated time points, euthanasia was performed via gradual CO_2_ displacement, following the guidelines outlined by the Institutional Animal Care and Use Committee (IACUC) and the American Veterinary Medical Association (AVMA). All animal procedures were reviewed and approved by the Thammasat University Institutional Animal Care and Use Committee (IACUC) under protocol number 002/2022. This study was conducted from April 2022 to June 2025 and all experiments were performed in strict compliance with the ARRIVE guidelines (https://doi.org/10.6084/m9.figshare.29598365) [27].

### Cryptococcal strains

The stock culture of *C. neoformans* H99 was preserved at −80 °C in the Department of Medical Technology, Faculty of Allied Health Sciences, Thammasat University. Prior to experimental use, the frozen stock was thawed and cultured on Sabouraud dextrose agar (SDA) (HiMedia) at room temperature for 48 h. Then, a single isolated fungal colony from the SDA was resuspended in Sabouraud dextrose broth (BD Difco) and cultured at 37 °C with shaking at 200 rpm for 24 h before infection [28].

### Mouse model of pulmonary *Cryptococcus* infection

Anesthetized mice received intranasal inoculation with 50 μl of either phosphate-buffered saline (PBS) or a suspension of *C. neoformans* (5 × 10^4^ CFU/mouse). For the *in vivo* treatment group, L-arginine (1.5 mg/g/day in sterile water) was administered daily via oral gavage [29]. At the designated timepoint, the lungs, spleens, and brains were aseptically harvested from euthanized mice. For colony-forming unit (CFU) analysis, the isolated organs were weighed and homogenized in sterile PBS containing 1 % penicillin and streptomycin. The homogenates were then serially diluted, plated on SDA, and incubated for 48 h at 30 °C. Fungal CFUs were subsequently enumerated, calculated, and expressed as CFU per gram of organ (CFU/g) [28].

### Analysis of mouse survival and clinical score

A total of 8 animals per group were included in the survival analysis and assessment of clinical parameters. Following intranasal infection with *Cryptococcus*, mice were observed throughout the infection period, and deaths were recorded for survival curve analysis. Body weight was measured, both before and during the infection daily, with results presented as the percentage change from baseline (Day 0). Additionally, clinical disease progression was quantitatively assessed by summing individual scores for specific observable signs, including ruffled fur, lethargy, hunched posture, tachypnea, and ataxia for each animal [30].

### Histopathological analysis

After euthanasia, lungs and brain were collected following perfusion and inflated with 4% paraformaldehyde. Organs were preserved in 10% neutral buffered formalin for storage, subsequently paraffin-embedded and sectioned. Organ sections were stained with hematoxylin and eosin (H&E) and Periodic acid-Schiff (PAS) to assess immune cell infiltration and the presence of *Cryptococcus* within the tissues. Images were captured using an Olympus DP21-SAL light microscope (Olympus, Canada).

### Analysis of serum arginase activity and arginine metabolites

Blood was collected from euthanized mice by cardiac puncture and non-hemolyzed serum was centrifuged, transferred into sterile tube, and stored at −80 °C for analysis of arginase activity and metabolites. Arginase activity was assessed by incubating 50 μL of serum with an activation solution at 56 °C for 10 minutes, followed by incubation with L-arginine at 37 °C for 2 hours [25]. The reaction was halted with an acid stop solution (H_2_SO_4_: H_3_PO_4_:H_2_O)and 9 % α-isonitrosopropiophenone (ISPF) in ethanol (Sigma-Aldrich) at 100 °C for 45 minutes. Arginase activity was quantified by measuring urea concentration via absorbance at 570 nm, with a urea standard curve to determine sample concentration (μg/mL).

For the analysis of serum metabolites in the L-arginine-arginase pathway, 25 μL of each serum sample was processed. The serum was mixed with 250 μL of 0.1% formic acid in isopropanol, then vortexed at 1,400 rpm for 10 minutes and centrifuged at 16,000 g at 4°C for 10 minutes. The resulting supernatant was collected and analyzed for arginine, citrulline, ornithine, and proline using an Ultimate 3000 Ultra-high performance liquid chromatography (UHPLC) tandem TSQ Quantis Triple Quadrupole Mass Spectrometer (Thermo Scientific, Waltham, MA, USA). Separation was performed on a Hypersil GOLD C18 column (1.9 μm particle size, 100 mm × 2.1 mm). The gradient program was carried out at a flow rate of 0.2 mL/min using 0.2% formic acid in Type I water and acetonitrile (ACN) as the mobile phase. Each compound was quantified and confirmed using its precursor mass and two product masses.

### BAL inflammatory cell analysis

Bronchoalveolar lavage (BAL) fluid and cells were collected and processed as previously described [26]. Briefly, after euthanasia, mouse lungs were instilled with 500 μL of ice-cold PBS containing 5 mM EDTA. BAL fluid was then collected via five subsequent washes. Following centrifugation, the supernatant was stored at −80 ^◦^C for subsequent cytokine analysis, as detailed previously [26]. The remaining BAL cells were enumerated and assessed for viability using trypan blue exclusion. These cells were then stained with fluorochrome-conjugated antibody to characterize lung inflammatory cell populations by flow cytometry. Specific cell populations identified included CD4^+^ T cells (CD3^+^ CD4^+^), dendritic cells (DC; CD11b^+^ CD11c^+^), alveolar macrophages (CD11c^+^ Siglec-F^+^), eosinophils (CD11c^−^ Siglec-F^+^), and neutrophils (CD11b^+^ Gr.1^hi^). Analysis was performed using BD FACSLyric cytometer (BD Biosciences) and FlowJo^TM^ software.

### Brain tissue collection and cytokine analysis

Whole brain tissue was aseptically collected from euthanized mice after cardiac perfusion with heparinized PBS. The brain tissue was weighed, immediately snap-frozen in liquid nitrogen, and stored at −80 °C until analysis. For lysate preparation, brain tissue was thawed and homogenized in modified RIPA buffer (50 mM Tris – HCl pH 7.4, 1% Triton X-100, 0.2% sodium deoxycholate, 0.2% sodium dodecyl sulfate, 1 mM EDTA) containing 1 mM PMSF (Phenylmethylsulfonyl fluoride, Sigma-Aldrich) and a protease inhibitor cocktail (Roche). Cytokine levels in brain lysates were quantified using the Bio-plex Pro™ Mouse Cytokine 23-Plex assay, following the manufacturer’s protocol (Bio-Rad Laboratory, USA). Fluorescent signals were measured using the Bioplex 200 System (Biorad), and data were processed with Bio-plex Manager 5 Software. Cytokine concentrations are reported in pg/ml [31].

### *In vitro* microglia treatment and infection

Mouse microglia cell line, BV2 cells, were cultured and maintained in complete RPMI medium (RPMI containing 10 % FBS, 1 % L-glutamine, and 1 % penicillin/streptomycin) [32]. 2 × 10^5^ cells were cultured in a 24-well culture plate containing complete RPMI for 24 h at 37^◦^C with 5% CO_2_. Before infection, the culture medium was replaced with serum-free medium for 2 hours, followed by activation with 1 mg/ml PMA for 30 minutes. The activated cells were then infected with cryptococci opsonized with 1 mg/ml anti-capsule mAb 18B7 at a multiplicity of infection (MOI) of 1:10. To determine the effect of L-arginine on cryptococcal proliferation and killing, the extracellular cryptococci were removed after 2 hours of infection. Subsequently, the *C. neoformans*-infected BV2 cells were cultured in SILAC DMEM Flex medium (DMEM lacking L-glutamine, L-lysine, and L-arginine; Gibco™) supplemented with 1 % L-glutamine, 1 % penicillin/streptomycin, 1% glucose, 1% NaNO_3_. In some experimental condition, L-arginine (400 μM) was added to the culture medium, and cells were incubated for 24 hours. For quantifying colony-forming units (CFU), infected cells at both 2 and 24 hours postinfection were lysed with sterile water and spread on SDA. For analysis of cryptococci at 24 hours, intracellular cryptococci (intracellular cryptococcal load) were recovered from the infected cells as described above and plated on SDA for CFU enumeration. These data were then used to calculate the intracellular proliferation rate (IPR) and intracellular killing rate of *Cryptococcus* at 24 hour postinfection by comparing the CFU at 24 hours with the initial intracellular CFU at 2 hours (IPR = intracellular CFU at 24 hpi./intracellular CFU at 2 hpi.; Percent killing at 24 hpi = [100 - (intracellular CFU at 24 hpi ×100)/intracellular CFU at 2 hpi.) [33,34].

### Brain endothelial cell culture and *in vitro* invasion assay

The murine brain endothelial cell line, bEnd.3, was prepared as previously described [25]. For the in vitro brain invasion assay, bEnd.3 cells were seeded at 6.6 × 10^4^ cells on a Matrigel-coated transwell insert (0.3-cm^2^, 8-μm pore size; BD Falcon, Corning) at the apical side. These inserts were then placed in a 24-well plate containing complete DMEM in the abluminal compartment. Cells were cultured until they reached 100% confluence, which was routinely monitored by Transendothelial electrical resistance (TEER). For macrophage coculture experiment, 6.6 × 10^4^ BV2 cells were added into the apical side with SILAC DMEM, with or without 400 μM L-arginine. Subsequently, the apical side of the insert was infected with *C. neoformans*, pre-opsonized with 40% fresh mouse serum, at a concentration of 4 × 10^5^ yeast cells/insert (corresponding to a multiplicity of infection of 6). For transendothelial analysis of cryptococcal invasion, the culture medium from the abluminal compartment of the insert (in a 24-well plate) was collected, serially diluted, and plated on SDA to determine CFU following incubation at 30 °C for 48 hours [25,35].

### Nitric oxide production assay

Nitric oxide synthase (NOS) activity was determined by measuring nitric oxide (NO) production. Nitrite accumulation in the sample, as an indicator of NO, was quantified using Griess reagent assay. Briefly, 100 μL of culture supernatant was combined with an equal volume of Griess reagent (1:1 mixture of 0.1% naphtylethylenediamine dihydrochloride and 1% sulfanilamide in 5% H_3_PO_4_). The mixture was then incubated for 10 minutes at room temperature. Absorbance was measured at 570 nm, and nitrite concentration was calculated from a nitrite standard curve [36].

### Quantitative RT-PCR

Total RNA from lung, brain and BV2 cells was extracted using a TRIzol™ reagent following the manufacturer’s protocol. Complementary DNA (cDNA) was then synthesized from the extracted RNA using oligodT and RevertAid Reverse Transcriptase (Thermo Scientific™). Gene amplification was performed using qPCRBIO SyGreen Mix (PCR Biosystems). The relative quantification of gene expression in the samples was conducted by normalizing the expression levels of interested genes to the endogenous actin (*Actb*) transcript. Results were calculated relative to the PBS control, as previously described [37,38]. The oligonucleotide primer sequences utilized in this study are listed in Supplementary Table S1.

### Statistical analysis

Each experiment was performed two to three times. Data were presented as mean values ± SD. Data were analyzed using the unpaired t-test (two-tailed) and one-way ANOVA with Turkey’s post hoc analysis. Kaplan – Meier survival curves and log-rank test were used for analysis of mouse survival. Statistical analyses were performed using GraphPad Prism 10 software. A *p* value < 0.05 was considered statistically significant.

## Results

### Serum L-arginine and its metabolite during *C. neoformans* infection

**Figure 1.**
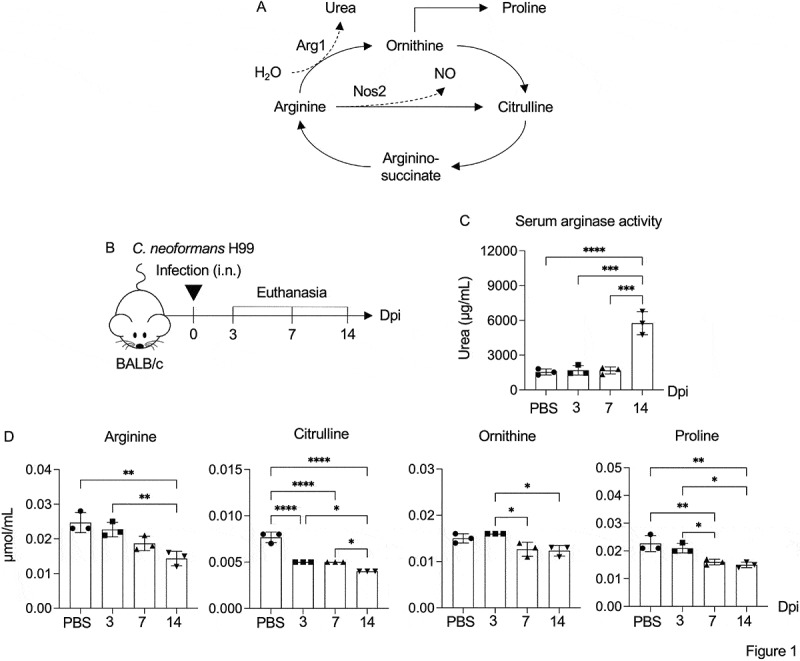
Serum arginine metabolites in high-virulence *C. neoformans* pulmonary cryptococcosis. (A) Schematic representation of the arginine metabolism, highlighting the division into two major pathways: the ornithine-proline pathway and the nitric oxide (NO)-citrulline cycle. (B-D) BALB/c mice were treated with PBS or intranasally infected with C. neoformans (H99) at 5 × 10^4^ yeast cells/mouse. At 3, 7, and 14 dpi., PBS-treated or *C. neoformans*-infected mice were euthanized, and serum was collected (B). Arginase enzyme activity in serum was determined by photometric measurement of urea concentration (C). Serum concentrations of arginine metabolites, including arginine, citrulline, ornithine and proline, were quantified by LC-MS/MS (D). Graphs show individual mice data with mean ± SD, representative of three independent experiments, with *n* = 3 mice per group. PBS-treated mice at 3 days postinfection were used as representative controls. Significance was determined using one-way anova with Tukey’s multiple comparisons test (*p < 0.05, **p < 0.01, ***p < 0.001, ****p < 0.0001).

During *C. neoformans* infection, previous studies have demonstrated that arginase activity is significantly induced in the lungs, which contributes to the activation of type 2 immune responses and the pathogenesis of cryptococcal disease [25,26]. Arginine is typically metabolized through the Arg1/Nos2 pathway, which is linked to downstream metabolites such as citrulline, ornithine, and proline (Figure 1(A)). However, the exact relationship between L-arginine metabolism and *Cryptococcus* infection is not yet fully elucidated. We first assessed the kinetic changes in systemic arginase enzyme activity and L-arginine metabolites in BALB/c mice infected with *C. neoformans* H99 at various stages of infection (Figure 1(B)). Serum samples were collected and analyzed for arginase activity, arginine, and their associated downstream metabolites using LC-MS/MS (Figure 1(C-D)). Compared to the PBS control group, mice infected with *C. neoformans* exhibited a notable elevation in serum arginase activity by 14 days postinfection (Figure 1(C)). Interestingly, all tested arginine metabolites underwent alterations during infection, each displaying unique patterns (Figure 1(D)). Specifically, a gradual depletion of arginine was observed, with its levels significantly reduced by 14 days postinfection. Although serum ornithine levels showed minimal changes, citrulline levels were markedly diminished at 3, 7, and 14 days, and proline levels were significantly lowered at 7- and 14-days postinfection. Collectively, these findings strongly suggest that diminished arginine availability during pulmonary *C. neoformans* infection, particularly in the late phase of infection, may contribute to the progression of cryptococcal disease.

### Expression of enzymes and transporters related to arginine metabolism during *C. neoformans* infection

**Figure 2.**
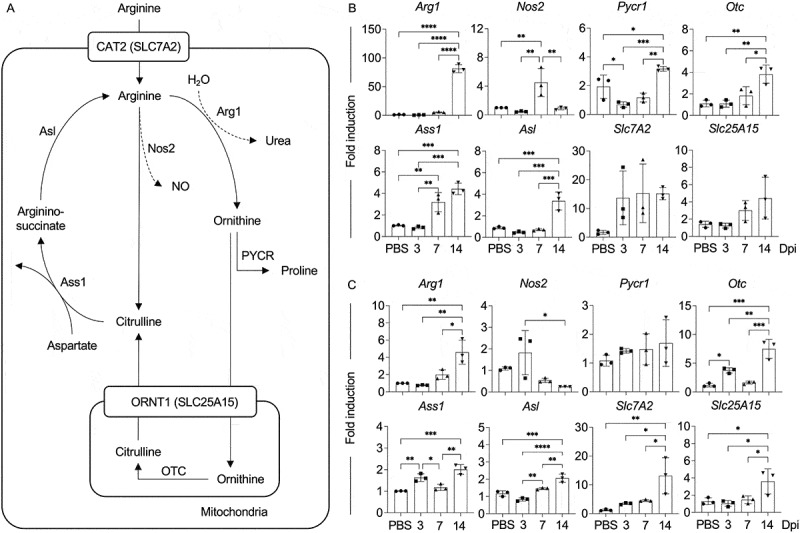
Gene expression of arginine metabolism enzymes and transporters during pulmonary *C. neoformans* infection. (A) Schematic representation of the cellular arginine metabolism. (B-C) BALB/c mice were treated with pbs or intranasally infected with *C. neoformans* (H99) at 5 × 10^4^ yeast cells/mouse. At 3, 7, and 14 dpi., lungs and brains were collected for gene expression analysis. Expression of genes encoding arginine metabolism enzymes and transporters, including *Arg1, Nos2, Pycr1, Otc, Ass1, Asl, Slc7A2*, and *Slc25A15* within lung (B) and brain (C) was determined by real-time PCR. Gene expression levels were normalized to endogenous actin (*Actb*) transcript levels, and fold induction was calculated relative to control (PBS) samples. Graphs show individual mice and mean ± SD, representative of three independent experiments, with *n* = 3 mice per group. PBS-treated mice at 3 days postinfection were used as representative controls. Significance was assessed using one-way anova with Tukey’s multiple comparisons test (*p < 0.05, **p < 0.01, ***p < 0.001, ****p < 0.0001).

Arginine metabolism primarily involves two major pathways: the ornithine-proline pathway and the nitric oxide (NO)-citrulline cycle, both of which are modulated by environmental signals [39,40]. L-arginine enters cells via the CAT2 (SLC7A2) transporter. In the ornithine-proline pathway, arginase-1 (Arg1) metabolized L-arginine, particularly during type 2 immune responses, to produce urea and ornithine. Ornithine is subsequently converted to proline by PYCR1, thereby supporting cellular proliferation and tissue remodeling. In contrast, nitric oxide synthase 2 (Nos2), activated by type 1 cytokines such as IFN-γ, competes with arginase for L-arginine to generate NO and citrulline. NO plays a key role in antimicrobial defense, while citrulline is recycled to sustain NO production. Additionally, citrulline can be synthesized from ornithine within mitochondria via ORNT1 (SLC25A15) and OTC (Figure 2(A)). Considering the gradual decrease in serum arginine levels observed during *C. neoformans* infection, we further examined the expression of key enzymes and transporters involved in arginine metabolism within the lung and brain, which are the primary sites of colonization and dissemination. In accordance with the changes in L-arginine metabolites, we found distinct expression patterns of arginine metabolism enzymes and transporters in the lungs (Figure 2(B)) and the brain (Figure 2(C)). In the lungs, genes encoding enzymes associated with arginine metabolism, specifically *Arg1, Pycr1, Otc, Ass1*, and *Asl*, were upregulated by 14 days postinfection (Figure 2(B)). Notably, *Nos2* expression was significantly induced only at day 7, without significant changes in the transporter genes (*Slc7A2* and *Slc25A15*) (Figure 2(B)). In the brain, the expression of *Arg1, Otc, Ass1*, and *Asl* was enriched by day 14, whereas *Nos2* showed a gradual downregulation (Figure 2(C)). Interestingly, the expression of transporter genes *Slc7A2* and *Slc25A15* was significantly induced in the brain at 14 days postinfection. These findings collectively suggest that *C. neoformans* infection can modify arginine metabolism specifically at the site of infection, particularly during the late phase of infection.

### L-arginine supplementation attenuates cryptococcal dissemination

**Figure 3.**
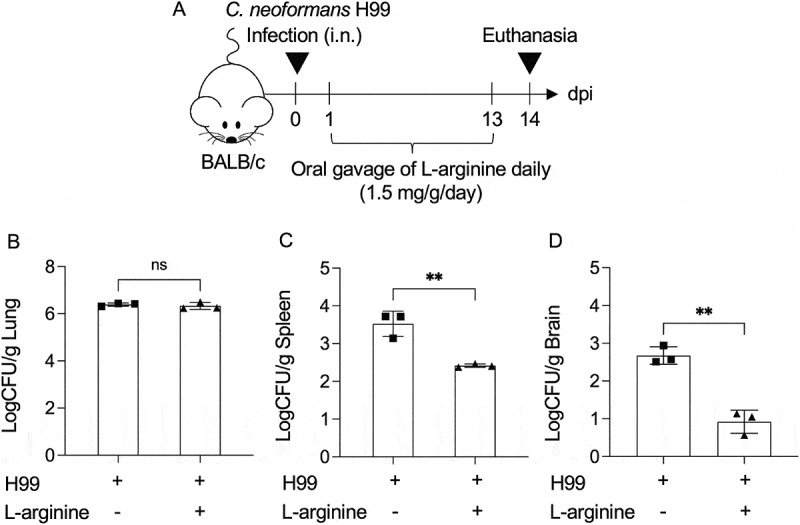
L-arginine administration reduces cryptococcal dissemination in mice. (A-D) BALB/c mice were intranasally infected with *C. neoformans* (H99) at 5 × 10^4^ yeast cells/mouse. Following infection, L-arginine dissolved in DI water was administered daily via oral gavage at a concentration of 1.5 mg/g/days (a). At 14 days postinfection, fungal burden within lung (B), spleen (C), and brain (D) was analyzed by CFU assay. Mice that received DI water were used as a control. Graphs show individual mice and mean ± SD is representative of three independent experiments, with *n* = 3 mice per group. Significance was determined using an unpaired t test (two-tailed). (ns; not significantly different, **p < 0.01).

Given the observed systemic reduction of L-arginine during cryptococcal disease progression, we investigated whether L-arginine supplementation confers beneficial effects in controlling *C. neoformans* infection. BALB/c female mice were intranasally infected with *C. neoformans* H99 and subsequently received daily oral gavage of L-arginine (1.5 mg/g) dissolved in sterile water. To assess the impact of L-arginine, fungal burdens in the lungs, spleen, and brain were quantified at 14 days postinfection, a time point when serum arginine levels were markedly reduced (Figure 3(A)). L-arginine supplementation significantly decreased fungal burden in the spleen and brain of infected mice, a reduction not observed in the lungs, when compared to controls receiving sterile water (Figure 3(B-D)). To address the potential influence of sex-specific immune responses, we determined the effect of oral L-arginine supplementation in BALB/c male mice. Similar to female mice, L-arginine treatment effectively reduced extrapulmonary fungal dissemination (Supplementary Figure S1). These findings suggest that L-arginine supplementation appears to be a promising strategy for managing cryptococcal infection, primarily through attenuating fungal dissemination.

### L-arginine enhances survival and lessens the severity of cryptococcal disease progression

**Figure 4.**
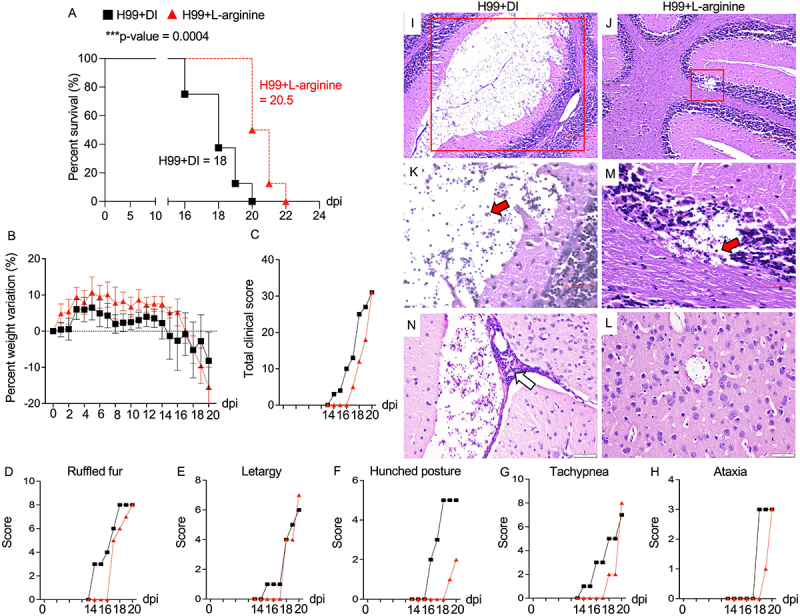
Effects of L-arginine treatment on mouse survival, clinical scores, and pathological responses during pulmonary *C. neoformans* infection *in vivo*. (A-F) BALB/c mice were treated with PBS or intranasally infected with *C. neoformans* (H99) at 5 × 10^4^ yeast cells/mouse. Following infection, L-arginine in DI water was administered daily via oral gavage at 1.5 mg/g/days. *C. neoformans*-infected mice (H99+DI and H99+L-arginine) were monitored for their survival and clinical presentation daily (8 mice per group). (A) Percentage of mouse survival during infection was analyzed using Kaplan – Meier survival curves, and p-value was obtained from a log-rank test (***p < 0.001). Percent weight variation (B), total clinical score (C), and individual clinical features such as ruffled fur (D), lethargy (E), hunched posture (F), tachypnea (G), and ataxia (H) were recorded. Significance of percent weight variation was determined using two-way anova. (I-L) at 20 days postinfection, brain from H99+DI and H99+L-arginine mice were collected for histopathological analysis. Representative brain images, stained with H&E, are shown at 10X ((I-J) and 40X (K-M) magnifications. Additionally, brain sections from H99+DI (N) and H99+L-arginine groups (L) were stained with PAS at 40X magnification. The area of cryptococoma in the brain was indicated by the red box. Red arrow indicates prominent colonization by *C. neoformans*. White arrow shows marked immune cells infiltrate indicating an active inflammatory response.

To further evaluate the role of L-arginine in modulating disease progression, we conducted a survival analysis in *C. neoformans*-infected mice with or without oral L-arginine administration. Mice receiving L-arginine demonstrated significantly prolonged survival compared to DI-treated controls (median survival: 20.5 days for H99 + L-arginine vs. 18 days for H99 + DI) (Figure 4(A)). Expanding on these findings, L-arginine-treated mice maintained better overall health, as indicated by a higher percentage of weight retention, particularly on day 16 postinfection (Figure 4(B)). We also evaluated clinical scores associated with disease severity, including ruffled fur, lethargy, hunched posture, tachypnea, and ataxia (Figure 4(C-H)). Interestingly, L-arginine-treated mice demonstrated a decreased total clinical score compared to DI-treated controls (Figure 4 (C)). Notably, improvements were predominately observed in clinical scores for ruffled fur, hunched posture, tachypnea, and ataxia, whereas no difference was detected in lethargy (Figure 4(D-H)). Histopathological analysis of the lungs and brain at 20 days postinfection revealed reduced inflammation in the lungs of L-arginine-treated mice (Supplementary Figure S2). More strikingly, treatment with L-arginine revealed a marked reduction in brain cryptococcomas compared to untreated group (Figure 4(I-M)), accompanied by decreased immune cell infiltration within brain lesions (Figure 4(N-L)). Collectively, these findings suggest that L-arginine supplementation appears to enhance fungal control in the brain and alleviate disease severity. This highlights L-arginine as a promising therapeutic adjunct in the management of *Cryptococcus* infection.

### L-arginine supplementation enhances brain protective cytokine response and tight junction-associated gene expression during *C. neoformans* infection

**Figure 5.**
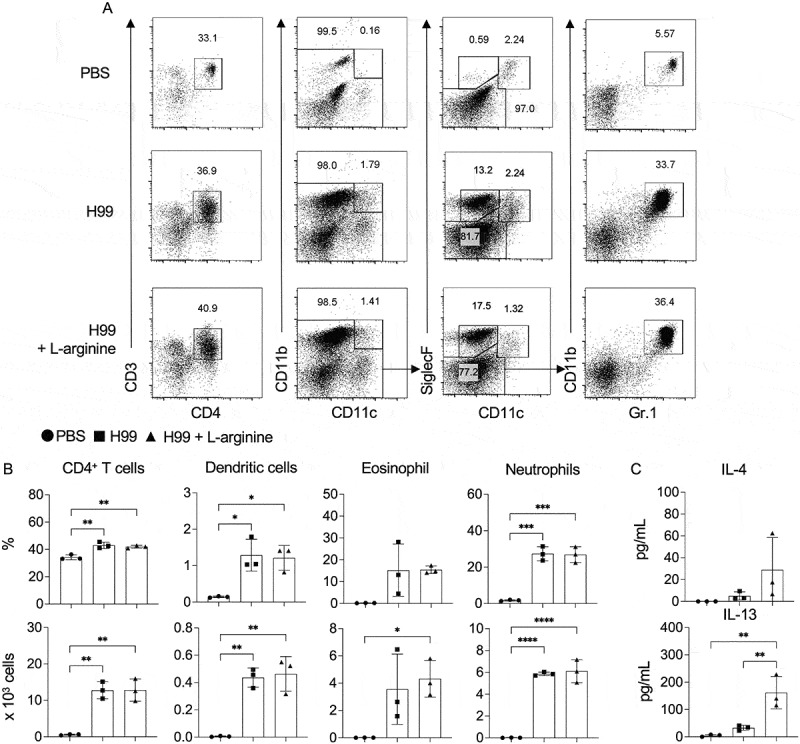
Analysis of pulmonary immune response in *C. neoformans*-infected mice treated with L-arginine. (A-C) BALB/c mice were treated with PBS or intranasally infected with *C. neoformans* (H99) at 5 × 10^4^ yeast cells/mouse, followed by daily oral gavage of L-arginine at 1.5 mg/g/days. At 14 days postinfection, PBS-treated and *C. neoformans*-infected mice, with or without L-arginine treatment, were sacrificed, and BAL inflammatory cells were counted and stained with fluorescence-conjugated Ab to CD3, CD4, CD11b, CD11c, Siglec-F and Gr-1. (A) representative plots indicate flow cytometry gating strategies for inflammatory cells: Th cells (CD3^+^ CD4^+^), dendritic cells (DC; CD11b^+^ CD11c^+^), eosinophil (CD11c^−^ Siglec-F^+^), alveolar macrophages (am) (CD11c^+^ Siglec-F^+^), and neutrophils (CD11b^+^ Gr-1^hi^). (B) Percentage and total cell number of inflammatory cells recovered from the BAL. (C) Cytokines production, including IL-4 and IL-13, in bal fluid was analyzed by ELISA. Graphs show individual mice and mean ± SD is representative of three independent experiments, with *n* = 3 mice per group. Significance was determined using one-way ANOVA with Tukey’s multiple comparisons test (*p < 0.05, **p < 0.01, ***p < 0.001, ****p < 0.0001).

We subsequently investigated the influence of L-arginine treatment on pulmonary cell infiltration and cytokine production. This focus was driven by the understanding that the induction of type-1/type-17 immune responses correlates with the control of *Cryptococcus* infection [37,41,42], whereas type 2 immune responses contribute to the pathogenesis and progression of cryptococcal diseases [37,43,44]. Indeed, there were no substantial differences in the overall quantity of inflammatory cells or the infiltration of leukocyte populations, including T helper cells, dendritic cells, eosinophils, and neutrophils, between infected mice administered sterile water or L-arginine (Figure 5(A-B)). Additionally, we quantified the concentrations of BAL fluid cytokines, including IFN-γ, IL-4, IL-13 and IL-17A, using ELISA. While IFN-γ and IL-17A levels remained undetectable, we observed a significantly increased concentration of IL-13 in the BAL fluid of *C. neoformans*-infected mice treated with L-arginine (Figure 5(C)).

**Figure 6.**
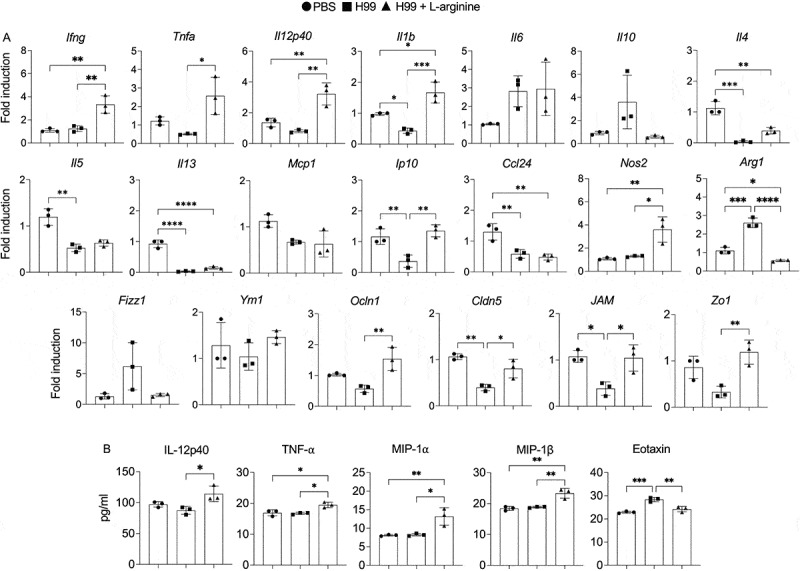
Oral administration of L-arginine increases type 1 cytokine expression in the brain of *C. neoformans*-infected mice. (a) At 14 days postinfection, the expression of brain genes, including *Ifng, tnfa, Il12p40, Il1b, Il6, Il10, Il4, Il5, Il13, Mcp1, Ip10, Ccl24, No2, Arg1, Fizz1*, *Ym1*, *Ocln1*, *Cldn5*, *JAM*, and *Zo1* was determined by real-time PCR. Expression levels of the target genes were normalized to endogenous actin (*Actb*) transcript levels, with fold induction calculated using the control (PBS) group as the baseline. (B) brain tissue lysates were analyzed for the production of IL-12p40, TNF-α, MIP-1α, MIP1β, and eotaxin using the Bio-plex mouse cytokine assays (Bio-Rad laboratories). Graphs show individual mice and mean ± SD is representative of three independent experiments, with *n* = 3 mice per group. Significance was determined using one-way ANNOVA with Tukey’s multiple comparisons test (*p < 0.05, **p < 0.01, ***p < 0.001, ****p < 0.0001).

L-arginine is also known to play a role in activating the inflammatory response in the brain [32,45]. We postulated that L-arginine treatment during infection may alleviate cryptococcal dissemination by modulating the inflammatory response in the brain. To address this hypothesis, we examined the expression of genes associated with fungal clearance in the brain. In contrast to infected mice receiving sterile water, we observed a significant elevation of inflammatory cytokines and chemokines, including *Ifng*, *Tnfa*, *Il12p40*, *Il1b*, and *Ip10*, in the brains of *C. neoformans*-infected mice treated with L-arginine (Figure 6(A)). Conversely, no significant differences were observed in the expression levels of *Il6*, *Il10*, *Il4*, *Il5*, *Il13*, *Mcp1*, and *Ccl24* between *C. neoformans*-infected mice with or without L-arginine treatment (Figure 6(A)). Given the observed upregulation of inflammatory cytokine gene expression, we further quantified cytokine levels in brain tissue lysates and found significantly increased levels of IL-12p40, TNF-α, MIP-1α, and MIP-1β, along with reduced eotaxin levels in *C. neoformans*-infected mice treated with L-arginine (Figure 6(B) and Supplementary Figure S3). L-arginine administration also resulted in a significant elevation in the expression of the M1 marker *Nos2*, accompanied by a reduction in the M2 marker *Arg1* (Figure 6(A)). Furthermore, the L-arginine-treated group exhibited elevated expression of tight junction genes, including *Ocln1*, *Cldn5*, *JAM*, and *Zo1*, compared to the untreated control (Figure 6(A)). Taken together, these data suggest that oral L-arginine treatment during *C. neoformans* infection promotes the expression of brain inflammatory cytokines, nitric oxide synthase, and tight junction-associated genes, which may contribute to protective immune responses and help prevent fungal dissemination within the brain.

### Effect of L-arginine on microglial response to cryptococcal infection

**Figure 7.**
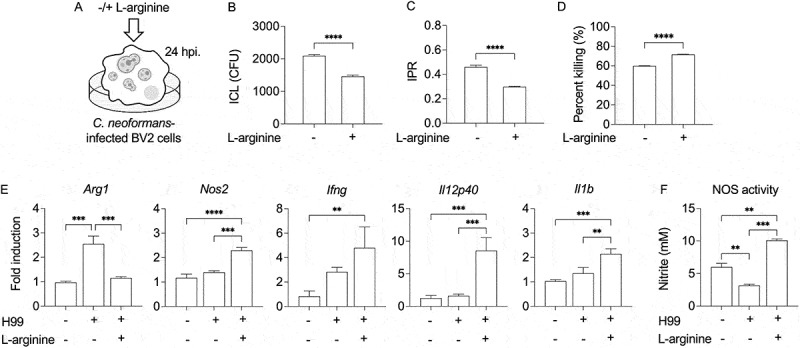
L-arginine enhances the antifungal function of microglia against *Cryptococcus in vitro*. (A-E) The BV2 microglia cells were infected with *C. neoformans* H99 at a 1:10 ratio. After 2 hours of infection, the infected BV2 cells were cultured in SILAC DMEM medium with or without L-arginine (400 μM) supplementation for 24 hours (A) to determine intracellular cryptococcal load (ICL) (B), intracellular proliferation rate (IPR) (C), and percent killing (D). Results are expressed as the means of three experimental repeats. Error bars denote mean ± SD. Significance was determined using an unpaired t test (two-tailed). ****p < 0.0001. (E) For gene expression analysis, the expression levels of *Arg1, Nos2, Ifng, Il12p40* and *Il1b* were determined by real-time RT-PCR. Expression levels of the target genes were normalized to endogenous actin (*Actb*) transcript levels, and relative quantification of the samples (fold induction) was calculated using data from noninfected cells as a baseline. (F) NOS activity in culture supernatant indicated by the nitrite concentration. Results are expressed as the mean of three experimental repeats. Error bars denote mean ± SD. Significance was determined using one-way ANOVA with Tukey’s post hoc analysis. **p < 0.01, ***p < 0.001, ****p < 0.0001.

Microglia, the brain-resident macrophages, act as the primary defense against invading pathogens in the brain. They exhibit antimicrobial functions by phagocytosing and killing cryptococci [46–48]. Conversely, microglia can also contribute to disease progression by enabling fungal persistence and dissemination [48–50]. Upon encountering *C. neoformans*, microglia can polarize into a pro-inflammatory M1 phenotype, linked to nitric oxide (NO) production and fungal clearance [46,51–53], or an anti-inflammatory M2 phenotype, which promotes tissue repair but may facilitate cryptococcal survival [54]. Given the observed elevation of inflammatory cytokines and M1 marker in the brain, we investigated whether L-arginine influences cryptococcal proliferation in microglia. We used the BV2 murine microglial cell line to assess L-arginine’s effect on microglial response to *C. neoformans* infection *in vitro*. BV2 cells were infected with *C. neoformans* H99 for 2 hours, followed by removal of extracellular cryptococci. These cells were subsequently cultured for 24 hours in SILAC DMEM, with or without L-arginine, and then examined for intracellular cryptococcal load (ICL), intracellular proliferation rate (IPR), and intracellular killing rate (Figure 7(A)). Compared to untreated cells, L-arginine treatment of *C. neoformans*-infected BV2 cells significantly reduced ICL (Figure 7(B)) and IPR (Figure 7(C)), while enhanced the intracellular killing rate (Figure 7(D)). Further analysis revealed that L-arginine treatment dramatically decreased *Arg1* expression, while augmenting gene expression of *Nos2* and other inflammatory cytokines, including *Ifng*, *Il12p40*, and *Il1b* (Figure 7(E)), and NOS activity (Figure 7(F)). These findings suggest that L-arginine augments microglial antimicrobial functions against *Cryptococcus* infection.

### L-arginine’s effect on cryptococcal invasion of brain endothelial cells *in vitro*

**Figure 8.**
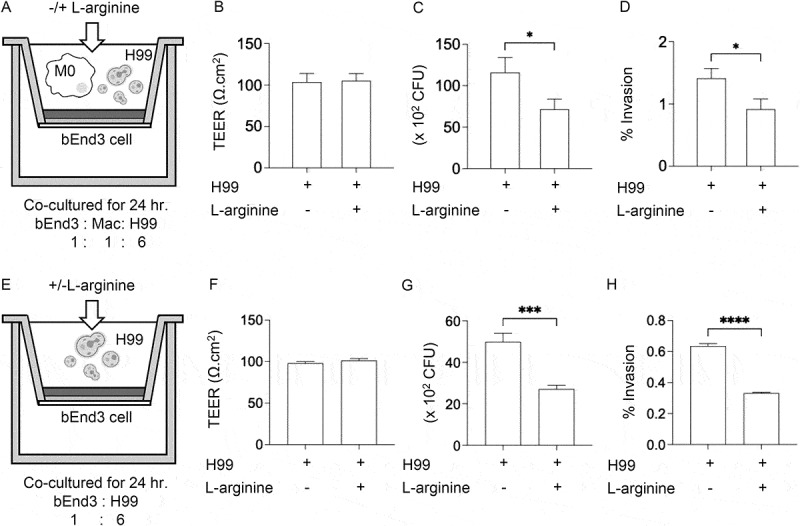
L-arginine treatment mitigates cryptococcal invasion in brain endothelial cells *in vitro*. Mouse brain endothelial cells were cocultured with opsonized *C. neoformans* H99, with (A-D) or without BV2 cells (E-H) on the apical side of a permeable insert in a 24-well plate in SILAC DMEM medium with or without L-arginine (400 μM). After 24 h, transendothelial electrical resistance (TEER) was measured (B, F). The culture medium from the abluminal part of the insert (in a 24-well plate) and apical (in an insert) was collected to determine the CFU (C, G) and percent invasion (D, H). Results are expressed as the means of three experimental repeats. Error bars denote mean ± SD. Significance was determined using an unpaired t test (two-tailed). *p < 0.05, ***p < 0.001, ****p < 0.0001.

Given that L-arginine reduced fungal dissemination to the brain and enhanced microglial antimicrobial functions, we investigated its effect on cryptococcal invasion of brain endothelial cells *in vitro*. The bEnd.3 murine brain endothelial cell line was cultured on permeable inserts within a Transwell plate to achieve intact barrier, and was cocultured with BV2 microglial cells and opsonized *C. neoformans* H99, with or without L-arginine (Figure 8(A)). We assessed barrier integrity via TEER measurement and analyzed cryptococcal invasion by quantifying CFU in the lower chamber and the percentage of fungal invasion. While L-arginine treatment did not affect the TEER values (Figure 8(B)), it diminished cryptococcal invasion into brain endothelial cells, as evidenced by reduced CFU in the lower chamber (Figure 8(C)) and lessened cryptococcal invasion rate (Figure 8D)). Given the observed impact of L-arginine on the expression of tight junction genes in the brain, we also investigated its direct effect on endothelial cell in regulating cryptococcal invasion (Figure 8E)). L-arginine treatment resulted in reduced CFU in the lower chamber and a decreased cryptococcal invasion rate, with no alteration in TEER (Figure 8(F-H)). Altogether, these data suggest that L-arginine modulates the brain’s inflammatory response *in vivo* and concurrently reduces fungal invasion into brain endothelial cells *in vitro*, potentially through coordinated regulation of microglial and endothelial cell functions.

## Discussion

L-arginine is a semi-essential amino acid that plays a crucial role in numerous biological processes. It serves both metabolic and immunomodulatory functions that are essential for tissue homeostasis, cellular communication, and host defense [55–57]. Our study provides novel insights into how L-arginine metabolism regulates immune responses during *Cryptococcus neoformans* infection. We observed a marked reduction in serum L-arginine and its related metabolites, suggesting a shift in host arginine metabolism. Oral L-arginine significantly lowered fungal burdens in the spleen and brain, demonstrating its role in mitigating cryptococcal dissemination, without affecting lung burden. Importantly, L-arginine treatment prolonged survival, improved clinical signs, and reduced brain cryptococcoma formation in infected mice. Furthermore, L-arginine supplementation improved microglial antifungal activity by promoting cryptococcal clearance and reducing fungal invasion in an in vitro blood – brain barrier (BBB) model. These findings underscore the therapeutic potential of L-arginine as an adjunctive treatment for cryptococcal meningitis or meningoencephalitis, where modulation of host metabolism may enhance immune control and improve disease outcomes.

Our data indicate that cryptococcal infection significantly altered serum L-arginine and its downstream metabolites (Figure 1(D)), particularly demonstrating depletion during the late phase of infection. This suggests a metabolic shift in L-arginine metabolism that may contribute to immune evasion and fungal persistence. Previous studies have shown an association between reduced plasma arginine level and the severity of several infectious diseases, such as Tuberculosis, Hand, Foot, and Mouth Disease (HFMD), HIV, as well as COVID-19 [58–61]. Therefore, the metabolic reprogramming of L-arginine during infections appears to be a common strategy exploited by pathogens to subvert host immunity. One of the key findings of our study is the tissue-specific modulation of enzymes and transporters related to arginine metabolism. In the lungs, an upregulation of *Arg1* and other enzymes linked to the ornithine-proline pathway may facilitate tissue remodeling and pathogen tolerance. Conversely, the transient increase in *Nos2* expression (Figure 2(B)) suggests a limited nitric oxide-mediated antimicrobial response during infection. In the brain, the significant induction of *Arg1* and its associated metabolic enzymes during the later stages of infection, along with the gradual downregulation of *Nos2* expression and increased expression of the arginine transporters *Slc7A2* and *Slc25A15* (Figure 2(C)), suggests enhanced arginine uptake, potentially compensating for systemic L-arginine depletion. This may reflect the pathogen’s strategy to evade immune responses, as nitric oxide produced by *Nos2* has antimicrobial properties [8]. Previous studies indicate that upregulated arginase activity in infections such as those caused by *Mycobacterium, Leishmania, Trypanosoma, Helicobacter, Schistosoma, Salmonella* and *Candida* contributes to pathogen persistence and disease progression [8,14,62–64]. The predominant activation of arginase results in L-arginine deprivation, a key mechanism responsible for the down-regulation of immune responses commonly observed in a wide range of pathological conditions, including chronic infections, cancer, autoimmune diseases, and sepsis [65–68]. During *Cryptococcus* infection, arginase-induced arginine deprivation may be recognized as an important mechanism facilitating the persistence of *Cryptococcus* within the CNS.

L-arginine is a well-known and widely utilized dietary supplement, popular for a variety of health benefits such as protein synthesis, muscle growth and repair, wound healing as well as immune system support [69]. Recent findings indicated that oral L-arginine supplementation shows promise for severe COVID-19 patients, leading to significant reduction in hospitalization duration and need for respiratory support after treatment [70]. In our study, oral L-arginine supplementation reduced fungal dissemination to the spleen and brain (Figure 3(B-D)), suggesting the L-arginine’s effect in restricting extrapulmonary fungal dissemination. Importantly, L-arginine-treated mice exhibited improved survival, reduced brain cryptococcomas, and ameliorated symptoms such as hunched posture (Figure 4(A-L)), implicating its role in modulating neuroinflammatory responses. Given the observed systemic L-arginine depletion during infection, these results support the hypothesis that restoring L-arginine levels rebalances host immunity and reduces fungal spread. Arginine is required for proper function of immune cells such as macrophage, neutrophil, T cells and B cells [9,71–74]. The administration of L-arginine may increase its serum level and promote protective function of immune cells. In tuberculosis, arginine supplementation has shown promise in improving clinical outcomes in HIV-negative TB patients by enhancing inflammatory responses [60,75]. Mechanistically, oral L-arginine administration in mice during *C. neoformans* infection led to a significant upregulation of protective inflammatory cytokines and chemokines in the brain (Figure 6, Supplementary figure S3), suggesting a shift toward a more effective antifungal immune response. Notably, elevated IL-13 levels in BAL fluid (Figure 5(C)) suggest that L-arginine treatment may also promote a localized type 2 immune response in the lung. These findings underscore the compartment-specific effects of L-arginine, with its dual potential to enhance protective immunity while also posing risks of localized immune dysregulation. Therefore, future translational efforts should focus on optimizing L-arginine dosing and delivery to maximize protective type 1 immune responses while minimizing type 2 skewing in the lung and mitigating CNS immunopathology.

L-arginine treatment increased *Nos2* and decreased *Arg1* expression in brain, suggesting a promotion of the protective M1 macrophage phenotype. Previous finding showed that L-arginine enhances antimicrobial activity of immune cells [76]. Our *in vitro* experiments with BV2 microglia confirmed that L-arginine enhances intracellular fungal killing and skews microglial polarization toward the M1 phenotype (Figure 7(A-F)), suggesting it strengthens microglial antifungal responses by modulating activation and promoting an effective immune response against cryptococcal infection. With the observed upregulation of tight junction genes by L-arginine supplementation *in vivo* (Figure 6) and reduced cryptococcal invasion across brain endothelial cells *in vitro* (Figure 8(E-H)), L-arginine may protect against cryptococcal infection in the CNS by enhancing microglial activation and maintaining endothelial cell integrity. Recent studies indicated that gut microbiota can utilize L-arginine to generate various bioactive compounds that regulate distal organ physiology and modulate host immune response during both homeostasis and disease [77,78]. Oral L-arginine administration has been found to reduce *Mycobacterium* pulmonary load by promoting inosine production from *B. pseudolongum* enriched in the gut [14]. Patients with cryptococcal meningitis exhibit significant gut microbiome alterations [79], suggesting that *C. neoformans* infection induces gut microbiota dysbiosis, potentially linked to disease progression. It remains unclear whether systemic depletion of L-arginine may influence gut microbiota-mediated contributions to host defense against cryptococcal infection.

In summary, our study reveals that *C. neoformans* exploits host L-arginine metabolism to evade immune responses, particularly in the CNS. Oral L-arginine supplementation in infected mice restores immune function, augments antifungal response, and alleviates clinical severity, highlighting its potential as an adjunctive treatment for cryptococcal infections. Future investigations should aim to elucidate the mechanistic link between L-arginine metabolism, gut microbiota, and neuroimmune modulation during cryptococcal disease progression, and establish a dose-response curve to define the therapeutic window and optimize its clinical application.

## Supplementary Material

Author Checklist_Oct_28.pdf

Revised_Supplemental Material_Oct_28.pdf

## Data Availability

The authors confirm that the data supporting the findings of this study are available within the article and its supplementary materials. The data that support the findings of this study are openly available in Figshare at https://doi.org/10.6084/m9.figshare.29598365 [27].
