# Supplementary material for: Oral L-Arginine treatment attenuates Cryptococcus neoformans extrapulmonary dissemination and disease progression
Source: Virulence. 2025 Nov 19;16(1):2591455. doi: 10.1080/21505594.2025.2591455 (PMC12645896; doi:10.1080/21505594.2025.2591455)
Supplement: Revised_Supplemental Material_Oct_28.pdf [file KVIR_A_2591455_SM8260.pdf]

Supplemental Material

Supplementary Table 1. Oligonucleotide primers used for Quantitative real-time PCR

Gene	Primer sequence (5' - 3')	
	Forward primer	Reverse primer
Arg1	TTGGGTGGATGCTCACACTG	TTGCCCATGCAGATTCCC
Nos2	GGAGTGACGGCAAACATGACT	TCGATGCACAACTGGGTGAAC
Fizz1	TACTTGCAACTGCCTGTGCTTACT	TATCAAAGCTGGGTTCTCCACCTC
Ym1	TCTCTACTCCTCAGAACCGTCAGA	GATGTTTGTCCTTAGGAGGGCTTC
Pycr1	CCCGAATATCCACGCTTTCT	ATGAAGCCTACGCTCATGTC
Otc	ACACTGTTTGCCTAGAAAGCC	CCATGACAGCCATGATTGTCC
Ass1	CTCGCAGACAGGTGGAGATT	GCCAGTGAATAGCAGGTGAG
Asl	ACTCTTGGAGGTGCAGAAGC	AGTAGCTCCCGGTCCACAC
Slc7A2	CCTTCATGGTCCCGTTCTTAC	TGTATGTCTGCGATGTGAGTG
Slc25A15	TCCACAGAAACCAGTAACGC	TCTGCATCTTCACCTTCATCG
Ifng	GATGCATTCATGAGTATTGCCAAGT	GTGGACCACTCGGATGAGCT
Tnfa	CAGGCGGTGCCTATGTCTC	CGATCACCCCGAAGTTCAGTAG
Il12p40	TTGGGTGGATGCTCACACTG	TTGCCCATGCAGATTCCC
Il1b	TTCAGGCAGGCAGTATCACTC	GAAGGTCCACGGGAAAGACAC
Il6	CCACGGCCTTCCCTACTTCA	TGCAAGTGCATCATCGTTGTTC
Il10	GGTTGCCAAGCCTTATCGGA	ACCTGCTCCACTGCCTTGCT
Il4	AGATCATCGGCATTTTGAACG	TGCAAGTGCATCATCGTTGTTC
Il5	CACACTGCGTCAGCCTACAGA	GCTTATTGAGGAGCTGAGCAACA
Il13	GCTTATTGAGGAGCTGAGCAACA	GCTTATTGAGGAGCTGAGCAACA
Ccl2(Mcp1)	ACTGAAGCCAGCTCTCTCTTCCTC	TTCCTTCTTGGGGTCAGCACAGAC
Cxcl10(Ip10)	AGTGCTGCCGTCATTTTCTG	ATTCTCACTGGCCCGTCA
Ccl24	CTCCAGAAGGCCCTCAGACTAC	GGGTCTTCATTGCGGTGG
Ocln1	GAGCTTACAGGCAGAACTAGAC	CAGCCATGTACTCTTCACTCTC
Cldn5	GGGTGGAACGCTCAGATTT	CTGGACATTAAGGCAGCATCTA
JAM	CACCGGGTAAGAAGGTCATTTA	GAACCTGTAGCACCTGAGTAAG
Zo1	GAATGATGGTTGGTATGGTGCG	TCAGAAGTGTGTCTACTGTCCG
Actb	GACGGCCAGGTCATCACTATTG	AGGAAGGCTGGAAAAGAGCC

Arg1, Arginase-1; *Nos2*, Nitric oxide synthase 2; *Fizz1*, Found in inflammatory zone 1; *Ym1*, chitinase-like 3; *Nos2*, Nitric oxide synthase 2; *Pycr1*, Found in inflammatory zone 1; *Otc*, Ornithine Transcarbamylase; *Ass1*, Argininosuccinate Synthase 1; *Asl*, Argininosuccinate Lyase; *Slc7A2*, Solute Carrier Family 7 Member 2; *Slc25A15*, Solute Carrier Family 25 Member 15; *Ifng*, Interferon (IFN) gamma; *Tnfa*, Tumor necrosis factor (TNF)-alpha; *Il12p40*, Interleukin-12 Subunit P40; *Il1b*, Interleukin-1 beta; *Il6*, Interleukin-6; *Il10*, Interleukin-10; *Il4*, Interleukin-4; *Il5*, Interleukin-5; *Il13*, Interleukin-13; *Ccl2*, C-C Motif Chemokine Ligand 2 (MCP-1); *Cxcl10*, C-X-C Motif Chemokine Ligand 10 (IP10); *Ccl24*, C-C Motif Chemokine Ligand 24; *Ocln1*, Occludin-1; *Cldn5*, Claudin-5; *JAM*, Junctional adhesion molecule; *Zo1*, Zonula Occludens-1; *Actb*, Beta-actin.

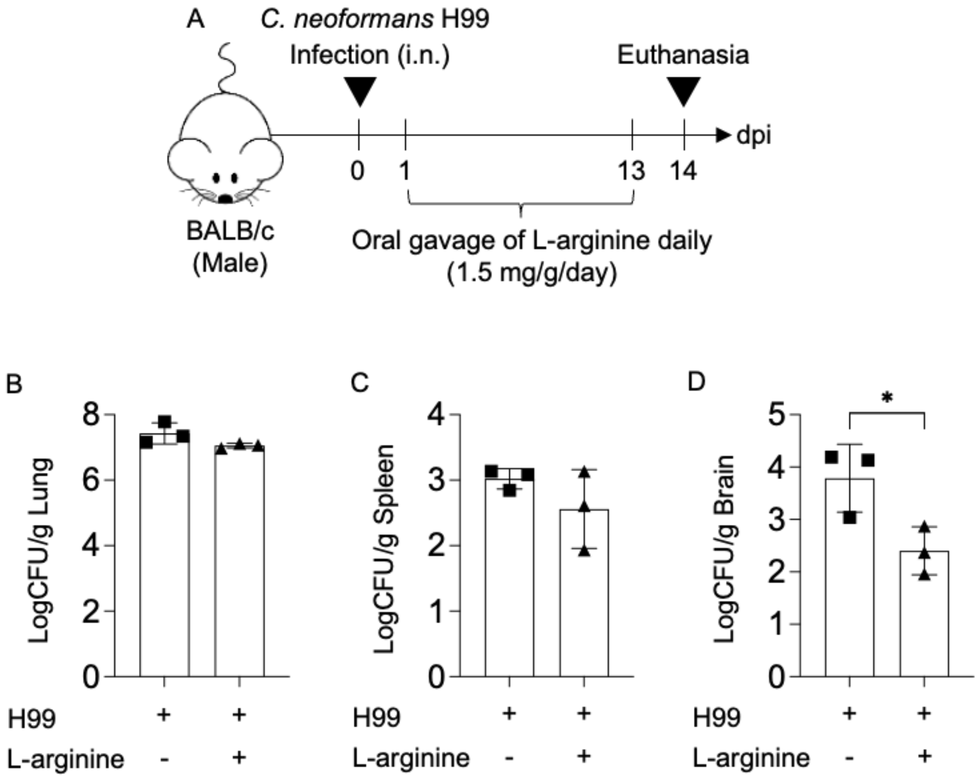

Supplementary Figure 1. Effect of oral L-arginine supplementation in *C. neoformans*-infected male mice. (A) BALB/c mice (male) were treated with PBS or intranasally infected with *C. neoformans* (H99) at 5 x 10^4 yeast cells/ mouse. Following infection, L-arginine in DI water was administered daily via oral gavage at 1.5 mg/g/days. At 14 days postinfection, fungal burden within (B) lung, (C) spleen, and (D) brain was analyzed by CFU assay. Mice that received DI water were used as a control. Graphs show individual mice and mean ± SD is representative of three independent experiments, with n = 3 mice per group. Significance was determined using an unpaired t test (two-tailed). (ns; not significantly different, *p < 0.05).

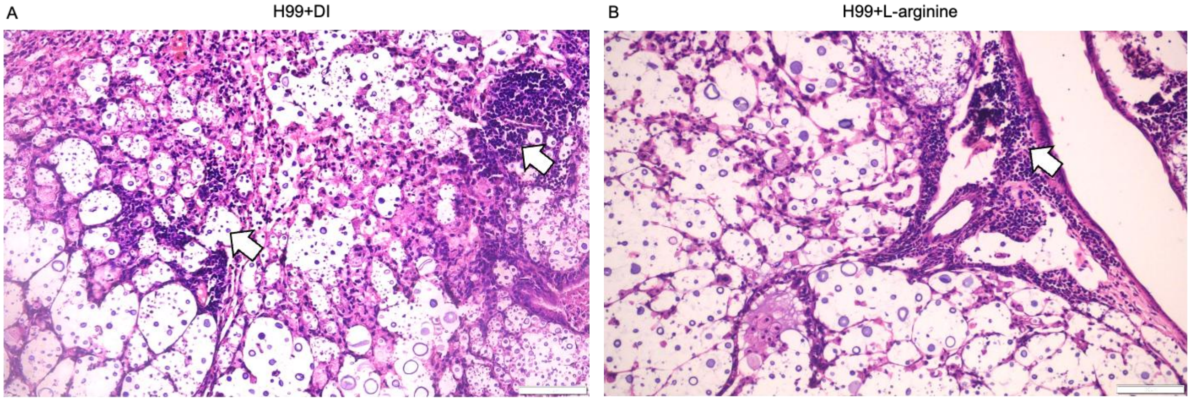

Supplementary figure 2. L-arginine treatment reduced pulmonary inflammation during *C. neoformans* infection (A-B) BALB/c mice were treated with PBS or intranasally infected with *C. neoformans* (H99) at 5 x 10^4 yeast cells/ mouse. Following infection, L-arginine in DI water was administered daily via oral gavage at 1.5 mg/g/day. (A) Lung section of H99+DI exhibits moderate histological changes, including inflammatory infiltrates and tissue alteration. (B) In contrast, lung tissue of H99+Arg shows only minimal histological changes. Both sections stained with H&E at original magnifications of ×20.

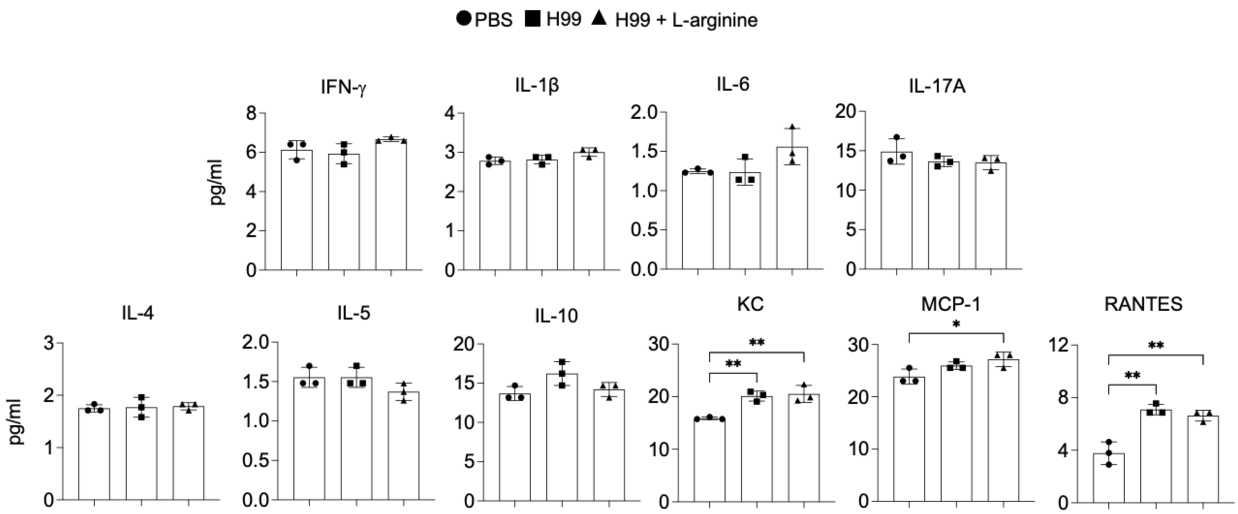

Supplementary figure 3. Assessment of Cytokine and Chemokine Profiles in Brain Tissue Lysates. BALB/c mice were treated with PBS or intranasally infected with *C. neoformans* (H99) at 5 x 10^4 yeast cells/mouse. Following infection, L-arginine in DI water was administered daily via oral gavage at 1.5 mg/g/day. Brain tissue lysates were analyzed for the production of IFN-γ, IL-1β, IL-6, IL-17A, IL-4, IL-5, IL-10, KC, MCP-1, and RANTES using the Bio-Plex Mouse Cytokine Assays (Bio-Rad Laboratories). Graphs show individual mice and mean ± SD is representative of three independent experiments, with n = 3 mice per group. Significance was determined using one-way ANOVA with Tukey's multiple comparisons test (*p < 0.05, **p < 0.01).
